# Design, fabrication, and characterization of a user-friendly microfluidic device for studying liver zonation-on-chip (ZoC)

**DOI:** 10.1007/s10544-025-00738-1

**Published:** 2025-02-14

**Authors:** Reza Mahdavi, Sameereh Hashemi-Najafabadi, Mohammad Adel Ghiass, Silmu Valaskivi, Hannu Välimäki, Joose Kreutzer, Charlotte Hamngren Blomqvist, Stefano Romeo, Pasi Kallio, Caroline Beck Adiels

**Affiliations:** 1 Biotechnology Department, Faculty of Chemical Engineering, Tarbiat Modares University, Tehran, Iran; 2 Biomedical Engineering Department, Faculty of Chemical Engineering, Tarbiat Modares University, P.O. Box, Tehran, 14115-114 IR Iran; 3 Tissue Engineering Department, Faculty of Medical Sciences, Tarbiat Modares University, Tehran, Iran; 4 Micro- and Nanosystems Research Group, Faculty of Medicine and Health Technology, Tampere University, 33720 Tampere, Finland; 5 Department of Physics, University of Gothenburg, 41296 Gothenburg, Sweden; 6 Department of Molecular and Clinical Medicine, Institute of Medicine, Wallenberg Laboratory, Sahlgrenska Academy, University of Gothenburg, Gothenburg, Sweden

**Keywords:** Liver zonation, Microfluidics, In-vitro, Ratiometric oxygen measurement, COMSOL simulation

## Abstract

Liver zonation is a fundamental characteristic of hepatocyte spatial heterogeneity, which is challenging to recapitulate in traditional cell cultures. This study presents a novel microfluidic device designed to induce zonation in liver cell cultures by establishing an oxygen gradient using standard laboratory gases. The device consists of two layers; a bottom layer containing a gas channel network that delivers high (cell incubator air, 19% oxygen) and low oxygenated (nitrogen) gases to create three distinct zones within the cell culture chamber in the layer above. Computational simulations and ratiometric oxygen sensing were employed to validate the oxygen gradient, demonstrating that stable oxygen levels were achieved within two hours. Liver zonation was confirmed using immunofluorescence staining, which showed zonated albumin production in HepG2 cells directly correlating with oxygen levels and mimicking *in-vivo* zonation behavior. This user-friendly device supports studies on liver zonation and related metabolic disease mechanisms *in vitro*. It can also be utilized for experiments that necessitate precise gas concentration gradients, such as hypoxia-related research areas focused on angiogenesis and cancer development.

## Introduction

**Fig. 1:**
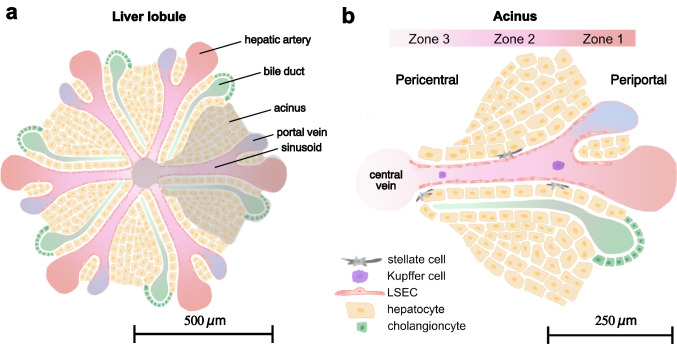
Schematic of the liver lobule and the influence of sinusoidal structure on hepatic zonation. (a) The liver lobule consists of hexagonal structures housing predominantly hepatocytes. The blood is supplied by the periportal vein and hepatic artery, providing nutrient and oxygen-rich blood respectively, which converge into the liver sinusoids. (b) The acinus is structured into three zones based on the hepatic function and metabolic activity. Endothelial cells (LSECs) line the vascular wall, shielding hepatocytes from shear stress while allowing constituents from the blood to diffuse into the tissue. As blood moves from the periportal side of the sinusoid toward the central vein, cellular consumption creates gradients of nutrients and oxygen along the sinusoid. These gradients are crucial in driving hepatic zonation, where hepatocytes display distinct functional behaviors depending on their location within the liver lobule. Additionally, other cell types such as stellate cells and liver-specific macrophages, known as Kupffer cells, each fulfill specific roles in both healthy and diseased liver tissue

The liver is composed of a set of highly dedicated cell types which organize into functional units known as liver lobules. The cells in the liver lobules specialize in essential tasks such as detoxification, bile production, protein synthesis, glucose regulation, and vitamin storage (Arias et al. 2020). Each liver lobule has six subunits (acinuses) where blood capillaries called sinusoids, are arranged radially and culminates at the central vein (Juza & Pauli, 2014), as depicted in Fig. 1. At the periportal end of the liver lobule, the hepatic artery and portal vein converge to supply the cells with blood rich in oxygen and nutrients. As these resources are consumed by the cells, concentration gradients will form along the sinusoids, with the highest concentration near the periportal end and the lowest near the central vein at the pericentral end. Consequently, cells encounter varying concentrations of nutrients, oxygen, and waste products based on their location, driving the phenomena of metabolic zonation within the liver lobule (Tomlinson et al. 2019). Metabolic zonation is a critical physiological feature that allows liver cells to exhibit diverse activities and characteristics depending on their position along the sinusoidal space. This zonation is often overlooked in conventional cell cultures, raising concerns about the accuracy of *in-vitro* models (Scheidecker et al. 2020).

The emergence of microfluidic technology has spurred efforts to develop *in-vitro* liver models that replicate the intricate microstructural liver functions found *in-vivo* (Leung et al. 2022). Microfluidics offers advantages such as precise spatiotemporal control over cell cultures by adjusting parameters like shear stress and availability of oxygen and nutrients, enabling cells to experience physiologically relevant environmental conditions (Rashidi et al. 2016; Vinci et al. 2011). Hence, *in-vitro* cultures in a microfluidic setting hold significant promise as tools for assessing drug efficacy and toxicity, effectively bridging the gap between preclinical and clinical testing (Materne et al. 2013; Zhang et al. 2018).

Incorporating zonation into an *in-vitro* liver model allows for the examination of specific niches in the context of disease or metabolic disruptions. For instance, metabolic dysfunction-associated steatotic liver disease (MASLD) predominantly affects hepatocytes in the pericentral zone, where the fatty acid beta-oxidation takes place (Brunt 2010). Conversely, hepatitis resulting from a yellow fever viral infection selectively spares hepatocytes around the periportal zone (Quaresma et al. 2006). Therefore, liver models with zonation features facilitate the investigation of liver diseases within the specific zone at the very location in which they are manifested. Additionally, these models allow for the exploration of inter-zonal communication between cells, providing insights into the mechanisms underlying liver diseases and metabolic disorders (Bale et al. 2014).

Different biomolecules display gradients within the liver acinus, where the concentration of dissolved oxygen plays a critical role in inducing zonation through β-catenin signaling (Kietzmann 2017; Tomlinson et al. 2019). In laboratory settings, scientists employ microfluidic liver-on-a-chip platforms to establish oxygen concentration gradients in cell cultures. Various methods, such as passive induction, the use of chemical oxygen scavengers, and the introduction of gaseous oxygen, are employed to generate gas gradients in these devices (Ghafoory et al. 2022; Kang et al. 2020; Kang et al. 2018). However, each method comes with its own inherent limitations. When employing passive induction, the generated gradient of nutrients and oxygen in the cell culture medium is solely driven by cellular metabolism, reflecting the natural zonation principles observed in liver-on-a-chip studies (Domansky et al. 2010; Lee-Montiel et al. 2017; Li et al. 2018). Nevertheless, the sensitivity of oxygen concentration to changes in flow rate poses challenges to controlling the microenvironment, and fluctuating cellular metabolism contributes to the issue. Adjusting the flow rate to modify the gradient may expose cells to varying shear stresses, potentially eliciting unwanted or detrimental cellular responses. Thus, the range of flow rate adjustments is limited and depends on cell type and microfluidic channel dimensions. Another approach to modulate the dissolved oxygen concentration in a microfluidic chip could be achieved by adding chemical oxygen scavengers such as sodium sulfate, cobalt nitrate, or deferoxamine (DFX) to the cell culture medium (Ghafoory et al. 2022; Kang et al. 2020; Kang et al. 2018). One major limitation with this approach is that scavengers alter the electrolyte balance of the cell culture medium and can adversely affect cells (Han et al. 2020). A more cell friendly option is to introduce gaseous oxygen through a permeable substrate like a polydimethylsiloxane (PDMS) membrane (Tonon et al. 2019; Tornberg et al. 2022). Typically, this non-invasive method necessitates access to gas facilities and large containers of costly oxygen mixtures, which are commonly available only in highly specialized mammalian cell culture laboratories.

In this study, we present a novel and user-friendly microfluidic device designed for 2D liver zonation-on-a-chip (ZoC) applications. Our ZoC device mimics liver zonation by incorporating an oxygen gradient and a parallel nutrient gradient, both aligned in the direction of the flow of cell culture medium. The oxygen concentration gradient in the ZoC cell culture chamber is formed utilizing an intricate microfluidic gas network where the gaseous oxygen is transferred to the cell culture chamber above through a PDMS membrane. This setup creates three distinct oxygen tension zones within the chamber, closely mimicking the *in vivo* environment. Concurrently, cells in these zones receive cell culture medium sequentially, replicating also the nutrient gradient observed *in vivo* within the acinus. To regulate the oxygen levels in the ZoC culture chamber, we utilize ambient incubator air and pure nitrogen as gas sources, both of which are readily accessible in standard laboratories. This approach not only allows for precise control of oxygen tension but also simplifies operational procedures for users. The microfluidic design, along with the distribution of glucose, oxygen, and shear stress, was simulated using COMSOL Multiphysics, both with and without cells in the ZoC device culture chamber. Experimental measurements using luminescence-based ratiometric oxygen sensors verified the oxygen tension levels. Finally, the viability of HepG2 cells was confirmed using the device, evaluating functionality based on spatially zonated albumin expression.

## Materials and methods

### Device design and fabrication

**Fig. 2:**
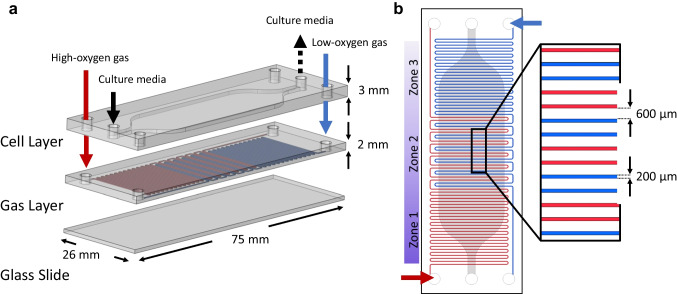
The computer-assisted design (CAD) of the chip layers and detailed gas channel network. **a** The split view, which consists of the upper cell chamber layer hosting the cells featuring an inlet (the solid black arrow) and an outlet (the dotted black arrow), a gas channel network layer of serpentine gas channels in the middle (the inlets for high-oxygen gas and low-oxygen gas are shown in blue and red arrows, respectively), and a glass slide at the very bottom. **b** The top view of the device, displaying the overlap of the gas and cell layers. It illustrates the positions of the gas channel inlet and outlets, demonstrating how these channels establish three distinct oxygenation zones within the cell chamber: high (zone 1, corresponding to the periportal zone), medium (zone 2) and low (zone 3, corresponding to the pericentral zone)

The device is fabricated in polydimethylsiloxane (PDMS) and comprises a dual-layer system designed to accommodate a 2D layer of perfused cells subjected to different oxygen levels. The lower layer (gas channel network) is approximately 2 mm thick, contains two non-intersecting gas channels measuring 0.2 mm in width and 0.1 mm in height. The upper layer (cell chamber) has a thickness of approximately 3 mm. Together, these layers contribute to an overall device thickness of about 5 mm bonded to a cover slide (see Fig. 2).

The gas channel network is supplied with two different gases: a high oxygenated gas (cell incubator air, 19% oxygen) and pure nitrogen gas (0% oxygen). This lower layer is plasma bonded facing the cover slide, leaving the gas to permeate through the PDMS material to reach the cells (see Fig. 2a). To achieve zonation, a suction is applied at one outlet with a vacuum pump (104 SA-VD (AC) Vibrating Diaphragm Pump, Schwarzer Precision) to guide the ambient air from the cell incubator into the chip at a flow rate of 2–3 mL/min, while nitrogen is introduced into the gas channel at the opposite side at an equal flow rate (see Fig. 2b). The design of the gas channel network has been carefully engineered to generate three distinct oxygen levels within the cell culture chamber, mirroring the *in-vivo* environment.

Initially, the cell chamber design and the corresponding photomask for the gas channel network pattern was generated using Corel DRAW. The gas layer was fabricated in two steps using soft lithography according to the methodology by Banaeiyan et al. (Banaeiyan et al. 2017). In short, a layer of the anti-photoresist SU-8 3050 was spin-coated for 30 s at 1000 rpm onto a silicon wafer, leaving a layer of 100 μm thickness of SU-8. The photomask was then placed on top the SU-8 coated wafer and the entire wafer was exposed to UV radiation for one minute (MA200 mask aligner, SUSS Microtech). The wafer was then developed using a developer solution (Micro Resist Technology GmbH), to remove the unexposed anti-photoresist, thereby revealing the patterned mold.

To create the cell chamber mold, a 1 mm sheet of poly(methyl methacrylate) (PMMA) was laser-cut based on the computer-assisted design (CAD) software design. The resulting structure was affixed to a flat glass surface using commercially available adhesive super glue (Henkel, Düsseldorf, Germany).

**Fig. 3:**
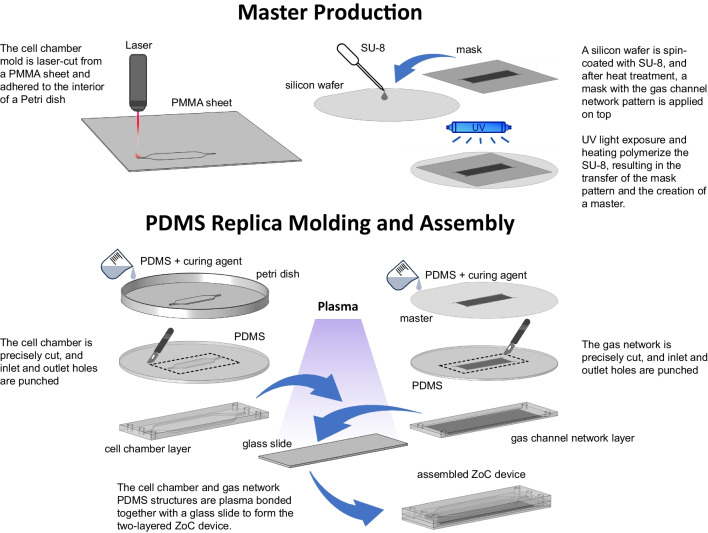
Fabrication process of the ZoC device with a gas channel network and cell chamber using soft lithography and laser cutting. A PMMA sheet was laser-cut to form the cell chamber mold meanwhile the mold for the gas channel network was fabricated using soft lithography. Here, an SU-8 anti-photoresist was spin-coated onto a silicon wafer and patterned via UV exposure to outline the gas channel network. Both molds were then used for polydimethylsiloxane (PDMS) replica molding and crosslinked with heat. Finally, the gas channel network and cell chamber layers were bonded together and attached to a glass slide, completing the assembly of the ZoC device

For the soft lithography procedure, a mixture of PDMS (Sylgard 184, Dow Corning, Midland, Michigan, US) and its corresponding crosslinker was prepared in a 10:1 (w/w) ratio. It is important to maintain the total height of the gas channel network within 2 mm ± 0.3 mm to ensure that the resulting oxygen gradient matches the simulation and remains consistent across experiments. After degassing, this mixture was poured onto both the cell and gas layer molds, and was left to incubate at 60°C overnight for crosslinking. After carefully removing resulting crosslinked PDMS from the molds, inlet and outlet holes were created using a 1.5 mm puncher (33-31AA-P/25, Miltex, Integra LifeSciences, Princeton, New Jersey, US). The two layers were precisely aligned and bonded together via air plasma treatment (18 W, 30 s; PDC-32G-2, Harrick Plasma, Ithaca, New York, US), followed by incubation at 60°C for 30 min. Subsequently, the device was plasma bonded onto a plain soda-lime glass slide (76 × 26 × 1 mm from Paul Marienfeld GmbH & Co. KG, Lauda-Königshofen, Germany) and incubated overnight at 60˚C before use. A schematic of the device fabrication is illustrated in Fig. 3.

### Numerical analysis

The numerical analysis was performed in COMSOL Multiphysics 6.0 finite element software (Burlington, MA, US) as a three-dimensional domain. The simulation model incorporated a coupled analysis of computational fluid dynamics (CFD) within the culture chamber and simultaneous glucose and oxygen mass transfers. While fluid flow and glucose distribution were confined to the culture chamber, the analysis of oxygen involved the entire device, including the PDMS bulk and gas network.

The flow simulation assumed Newtonian incompressible laminar flow with a flow rate of 0.5 µL/min and no-slip boundary conditions at the channel walls. The outlet pressure of the device was set to atmospheric pressure. Density and dynamic viscosity values were assigned as 1.009 g/cm^3^ and 0.93 mPas, respectively, based on the properties of the DMEM culture media supplemented with 10% FBS at 37°C (Poon, 2020).

The fluidic behavior of the flow in the laminar regime was simulated under the assumptions of constant fluid density and viscosity throughout the culture chamber, and it coupled the mass conservation and Navier–Stokes as the governing equations:

$$\rho \nabla \cdot u = 0$$

and

$$\rho \left(\frac{\partial u}{\partial t}+ u\nabla u\right) = -\nabla p + \mu {\nabla }^{2}u + F,$$

where $\rho$ is the density of the fluid, $u$ is the velocity vector, $t$ is time, $p$ is the pressure, $\mu$ is the dynamic viscosity, $\nabla$ is the gradient operator, ${\nabla }^{2}$ is the Laplacian operator, and $F$ is any external force acting on the fluid.

We utilized the convection–diffusion equation to simulate the mass distribution of oxygen and glucose:

$$\partial C/\partial t + \nabla \cdot (uC) = D {\nabla }^{2}C.$$

Here, $C$ is the concentration of the species being transported, $t$ is time, $u$ is the fluid velocity, and $D$ is the diffusion coefficient set to 6.16 × 10^−10^ m^2^/s and 2.69 × 10^−9^ m^2^/s, for glucose and oxygen in the cell culture medium at 37°C, respectively (Bavli et al. 2016; Place et al. 2017).

For modeling the oxygen distribution, the oxygen diffusion coefficient in PDMS was set to 3.25 × 10^−9^ m^2^/s (Markov, Lillie, Garbett, & McCawley, 2014), with saturated oxygen concentration applied at the boundaries exposed to the atmosphere, measuring 1.8 mol/m^3^. The interface between the cell culture medium and PDMS was characterized using a partition coefficient of 10 (Shiku et al. 2006), and the cellular oxygen consumption rate (OCR) was modeled using the Michaelis–Menten equation:

$$OCR =\frac{{qN}_{t}}{{A}_{t}} \left(\frac{C}{\left({K}_{m} + C\right)}\right),$$

where ${K}_{m}$ is the Michaelis–Menten constant equal 6.3 × 10^–3^ mol/m^3^ (Wagner et al. 2011), $q$ is the OCR for each cell, ${N}_{t}$ is the total number of cells, ${A}_{t}$ is the cell chamber area, and $C$ is the local concentration of oxygen at the lower surface of the culture chamber. The cellular OCR, denoted as $q,$ was set to match that of hepatocytes, specifically 3.5 × 10^–16^ mol/s (Wagner et al. 2011). Also, a Heaviside function was used to account for cell necrosis resulting from hypoxia. The critical oxygen concentration at which the cells undergo necrosis was defined as 1.0 × 10^–4^ mol/m^3^ (Buchwald 2009). For oxygen concentration simulation in the whole device, two scenarios were considered: one with cells present (400k cells/mL of seeding density) and another without cells. In each scenario, both static and time-dependent modes were employed to analyze the equilibrium and dynamic behavior of the system, respectively. A flow rate of 0.5 µL/min was selected for both scenarios.

At the cell chamber bottom surface, a negative flux was introduced into the system based on the calculated glucose consumption rate of the HepG2 cell line. The simulation of glucose distribution was performed in conjunction with the oxygen distribution study within the cell chamber. Initially, glucose concentration at the entrance of the cell chamber was set to the glucose content of the culture media at 5.5 mM. To estimate glucose consumption, we utilized the glucose uptake rate for the hepatic cell line, which is 2.4 × 10^–9^ mol/min per million cells (Bavli et al. 2016). This rate was then normalized based on the total cell count and culture area within the device.

### Ratiometric oxygen sensing

To analyze the microfluidic spatiotemporal oxygen pattern, a ratiometric 2D oxygen imaging system was utilized, following the methodology outlined by Ungerböck et al. (Ungerböck et al. 2013). This system utilizes the red and green channels of an RGB camera as the oxygen-sensitive, and reference channel, respectively. Oxygen levels were measured using Platinum (II)−5,10,15,20-tetrakis-(2,3,4,5,6-pentafuorphenyl)-porphyrin (PtTFPP) (Livchem Logistics GmbH, Frankfurt, Germany) as the oxygen-sensitive dye and Macrolex Fluorescent Yellow (MFY) (Livchem Logistics GmbH, Frankfurt, Germany) as the reference dye. Oxygen sensing films with a thickness of approximately 14 µm were knife-coated on glass plates, as described in detail by Tornberg et al. (Tornberg et al. 2022).

A CCD RGB camera (Axiocam 503) mounted on an inverted fluorescence microscope (Zeiss Axio Observer Z1) was used for the oxygen imaging. Images were acquired with a 2.5X objective (2.5x/0.06) and using an FITC filter set consisting of a bandpass 450–490 nm excitation filter, a 510 nm dichroic mirror and a long pass 515 nm emission filter. A 455 nm LED source (M455L4-C4, 690 mW) was used for the illumination.

The sensor material was blended with polystyrene (PS) that has a substantially lower oxygen permeability compared to PDMS, and hence could impair the oxygen diffusion within the device. Therefore, instead of covering the entire cell culture surface, small sensor sections were incorporated on the bottom of the cell chamber. These sensor sections, each measuring 1.5 mm in diameter, were created by first detaching the knife-coated film from the glass plate by immersing the oxygen sensing plate in deionized water, and then placing the detached sensor film on a PDMS membrane, and finally cutting circular pieces from a sensor film using a biopsy puncher. This approach not only ensures the effective functioning of the device gas exchange with real-time oxygen sensing, but also allows for microscopy imaging of the remaining cell culture areas, as the rest of the cell culture surface is optically transparent [28]. During the device fabrication process, ten sensor film sections were gently bonded to the bottom of the culture chamber using a plasma bonding. To ensure homogeneity for cell attachment, this surface received a thin layer of spin-coated PDMS (approximately 10 µm) using 0.5 mL PDMS at 1000 rpm for three minutes. The fabrication process was finalized by incubating the device at 70°C for 15 min to polymerize the spin-coated PDMS.

### Oxygen measurement and calibration

Prior to the measurement, the high-oxygen gas (19% oxygen) was infused into both gas channels for a minimum of one hour to establish equilibrium within the device. Subsequently, one of the gas channels was switched to 0% oxygen (i.e., nitrogen gas), while the other continued to receive a continuous flow of high-oxygen gas. Imaging started immediately, capturing fluorescence images at 10-min intervals. At the end of the experiment, a sodium sulfite solution (Sigma Aldrich, Missouri, USA) with a concentration of 10 mg/mL was injected into the cell chamber to scavenge oxygen and ensure 0% oxygen concentration for calibration purposes.

For image analysis, a customized MATLAB program was applied. Within each defined region of interest (ROI), we computed the ratiometric parameter value *R* by dividing the red channel mean value by the green channel mean value. Subsequently, the ratiometric parameter values for each gas mixture were subjected to fitting using the simplified two-site Stern–Volmer equation (Demas et al. 1995):

$$\frac{R}{{R}_{0}} = \frac{{f}_{1}}{1 + {K}_{sv}\left[{O}_{2}\right]} + {f}_{2}.$$

Here, $R$ represents the ratio of the dye emissions at an oxygen concentration of $[{O}_{2}]$, and ${R}_{0}$ represents the ratio of the dye emissions in the absence of oxygen. Additionally, ${f}_{1}$ represents the fraction of dye molecules that are available to quenching and is associated with the Stern–Volmer coefficient ${K}_{sv}$, while ${f}_{2}=1-{f}_{1}$ represents the fraction of dye molecules that cannot be quenched. The fitting procedure provided estimations for ${f}_{1}$ and ${K}_{sv}$, which were subsequently used to determine the oxygen concentration during the measurements. Calibration procedures were conducted for each ROI prior and after each measurement for 19% and 0% oxygen, respectively.

### Cell culture

HepG2 cells (ATCC, Menassas, VA, USA) were cultured in T-75 flasks using Minimum Essential Media/Earle’s Balanced Salt Solution (MEM/EBSS) medium (SH30244.01, HyClone, GE Healthcare, Chicago, Illinois, USA) supplemented with 10% Fetal Bovine Serum (FBS) (SV30160.03, HyClone, Logan, UT, USA), 1% penicillin–streptomycin (SV30010, HyClone), 1% L-glutamine, 1% sodium pyruvate (BE13-115E, Lonza, Basel, Switzerland), and 1% non-essential amino acids (SH30238.01, HyClone). The cells were maintained in a humidified incubator at 37°C with 5% CO_2_ until they reached a confluency of 70–80%. Subsequently, the cells were detached from the flasks using TrypLE Express (Gibco, Thermo Fisher Scientific, Waltham, MA, USA), centrifuged at 1000 rpm for 2 min and the supernatant was discarded. The cells were resuspended in cell culture medium to achieve a final concentration of 4 × 10^5^ cells/mL. Cells used for culturing in the device did not exceed passage number 25. The cells were seeded at a number and density that align with the oxygen consumption rates used in the numerical simulations, while ensuring they remain below the recommended 80–85% confluency after 26 h of culture. Additionally, to enable effective albumin staining and analysis, the goal was to have individual cells rather than clusters of aggregated cells by the end of the experiment.

To ensure sterility, the devices were sterilized using 70% ethanol, followed by rinsing with sterile de-ionized water. The cell chamber was coated with poly-Lysine hydrobromide (Sigma, 100 μg/ml) and incubated at room temperature for two hours. Subsequently, a HepG2 cell suspension (400k cells/mL) was introduced into the device via a micropipette and allowed to attach for two hours. The device was then connected to a syringe pump (CMA 400, CMA Microdialysis, Kista, Sweden) set at a flow rate of 0.5 µL/min. After connecting the gas lines to the device, it was placed in a humidified incubator for 24 h at 19% O_2_, 5% CO_2_ and 37°C.

### Cell viability and imaging

To assess the viability of HepG2 cells within our microfluidic device, we utilized a live/dead staining assay (L3224; Thermo Fisher Scientific). This assay utilized a mixture of calcein-AM (1:1000 v/v), ethidium homodimer-1 (1:1000 v/v), and Hoechst (1:2000 v/v) in phosphate-buffered saline (PBS). These stains labeled living cells, dead cells, and total cell nuclei, respectively. The cells were exposed to the staining solution for 15 min at 37°C in a light-protected environment and then rinsed with PBS containing calcium and magnesium. Imaging was carried out using a Leica fluorescence microscope (DMI6000B microscope, Leica Microsystems, Wetzlar, Germany). For quantitative analysis, the acquired images were processed using ImageJ software. After applying the appropriate threshold, the numbers of living, dead, and total cells were determined based on the green, red, and blue channels, respectively. The percentage of living cells was calculated using the following formula:

$$Viability \left(\%\right)=\frac{total\;cells- dead\;cells}{total\;cells }\times 100$$

### Albumin immunofluorescence staining

After a 24-h exposure to the oxygen gradient, the microfluidic device was disconnected from the syringe pump. To access the cell chamber area, the cell chamber was opened from the top by cutting around the edges of the chamber walls with a scalpel. A washing procedure was then carried out using PBS at 37°C. Subsequently, the cells were fixed using 4% formaldehyde for 15 min at room temperature, followed by three consecutive washes with PBS. The cells were then permeabilized with 0.25% Triton X-100 in PBS for five minutes, followed by three additional PBS washes. To block non-specific antibody binding, a solution of dried milk (5 mg/mL) was applied. The cells were then incubated overnight at 4°C with a diluted primary antibody solution specific to albumin (1:200 goat anti-albumin, Bethyl Laboratories, A80-129A) in PBS. The following day the secondary antibody (1:2000 Donkey anti-goat, Alexa Fluor 594, Thermo Fisher Scientific, A-11058) diluted in 1% BSA was added to the samples. After two hours at room temperature in the dark, the samples were washed three times with PBS. To counterstain the cell nuclei, Hoechst (1:2000, 33,258; Thermo Fisher Scientific) was applied for 15 min, and the cells were subsequently washed with PBS. The imaging process was performed as previously detailed in the earlier section.

### Quantification of immunofluorescence staining

Automated fluorescence imaging involved capturing 36 images of each zone, arranged in a square array at 20× magnification. Prior to image acquisition, meticulous positioning and focusing were applied to each imaging site. To avoid image overlap of the readouts and minimize the risk of nearby imaging sites being exposed to excitation light, which might lead to bleaching and impact results, an offset equal to 50% of the field of view size was chosen between each image. The image scanning started from one corner, moving back and forth to cover all the designated imaging sites.

Images were saved in the raw format to facilitate subsequent image channel separation through Leica Application Suite X (LAS X) software. For immunofluorescence staining quantification, each image was converted to 8-bit and grayscale using ImageJ, with the greyscale value of each fluorescent channel extracted. To normalize the signal, the red signal value (indicative of albumin staining) was divided by the blue signal value (representing Hoechst staining of the cell nuclei) and presented as relative fluorescent units (RFU) for comparative analysis.

### Statistical analysis

The results were derived from a minimum of three independent experiments (n = 3), and values are presented as the mean ± standard deviation (SD). The statistical method employed for hypothesis testing was a one-way Analysis of Variance (ANOVA), with a significance level set at p < 0.05 to denote statistical significance.

## Results

### Numerical analysis and calibration of device performance

**Fig. 4:**
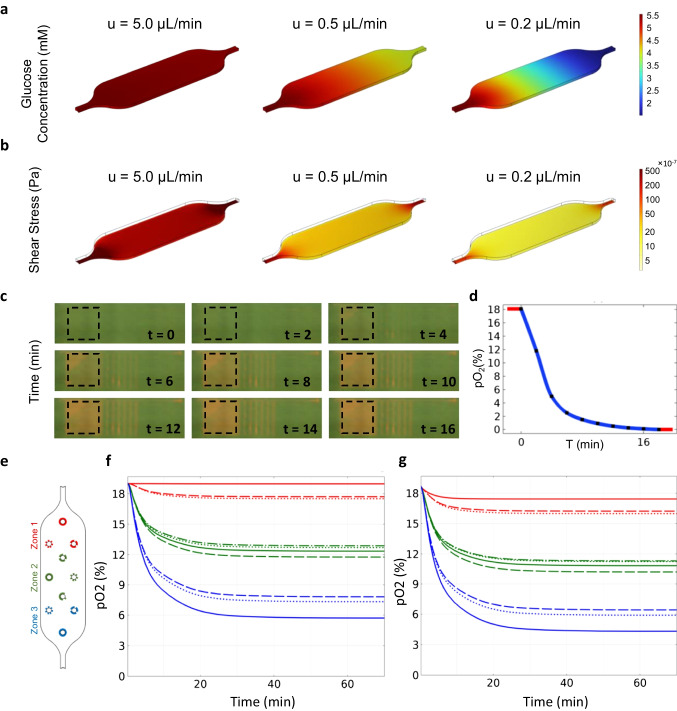
Simulation results of the ZoC device. **a** The glucose distribution at three different flow rates of 5.0, 0.5, and 0.2 µL/min is presented. At 5.0 µL/min, the glucose concentration at the outlet is 5.3 mM. For 0.5 µL/min, the concentration decreases to 4.2 mM, and at 0.2 µL/min, it drops further to 2 mM. **b** The shear stress values experienced by the cells in the culture chamber at flow rates of 5.0, 0.5, and 0.2 µL/min. At a flow rate of 5.0 µL/min, the shear stress is 2 × 10^−5^ Pa. This value decreases to 3.4 × 10^−6^ Pa at 0.5 µL/min, and further reduces to 1.1 × 10^−6^ Pa at 0.2 µL/min. **c** Timelapse micrographs of gas channel and sensor response as the gas flow is initiated (i.e., as the gas composition in the nitrogen gas channel shifts from 19% oxygen to 0% oxygen while the gas composition in the oxygen gas channel is kept constant at 19% oxygen). **d** The measured oxygen concentration from the marked area in panel c as a function of time. The curve uses red to represent the anoxic condition (0% oxygen) at the end of the experiment, which is caused by the addition of an oxygen scavenger, and the initial condition of 19% oxygen. **e** ROIs corresponding to the locations of the sensor sections used in the experimental oxygen measurement (Fig. 5). **f** Simulated oxygen concentration data from each ROI in e, depicting the oxygen dynamics within the device and recorded after simulating the initiation of the gas flow. The blue, green and red curves correspond to zone 1, zone 2 and zone 3, respectively. **g** Simulated oxygen concentration data from each ROI in e, also including the cells’ oxygen consumption

The numerical analysis of the device performance was focused on the zonation regarding the distribution of glucose and oxygen, as well as on the shear stress within the device. Since the presence of cells contribute to the zonation, the CFD simulations were performed both with cells and in a cell-free scenario. Glucose distribution is shown in Fig. 4a at three flow rates: 5.0, 0.5, and 0.2 mL/min. Figure 4a illustrates the effect of these flow rates on glucose distribution within the device. It is observed that at 5.0 µL/min, the glucose concentration does not undergo significant changes. However, at a flow rate of 0.5 µL/min, the glucose concentration decreases slightly, from 5.5 mol/m^3^ at the inlet to 4.2 mol/m^3^ at the outlet, when accounting for cellular metabolism. At the very low flow rate of 0.2 µL/min, the cells consume all the available nutrients, and the glucose concentration drops to 2 mol/m^3^. At a flow rate of 0.5 µL/min and a seeding density of 400k cells/mL, most of the culture area experienced shear stress around 2.8 × 10^–6^ Pa. Figure 4b shows the simulated shear stress profile for different flow rates.

**Fig. 5:**
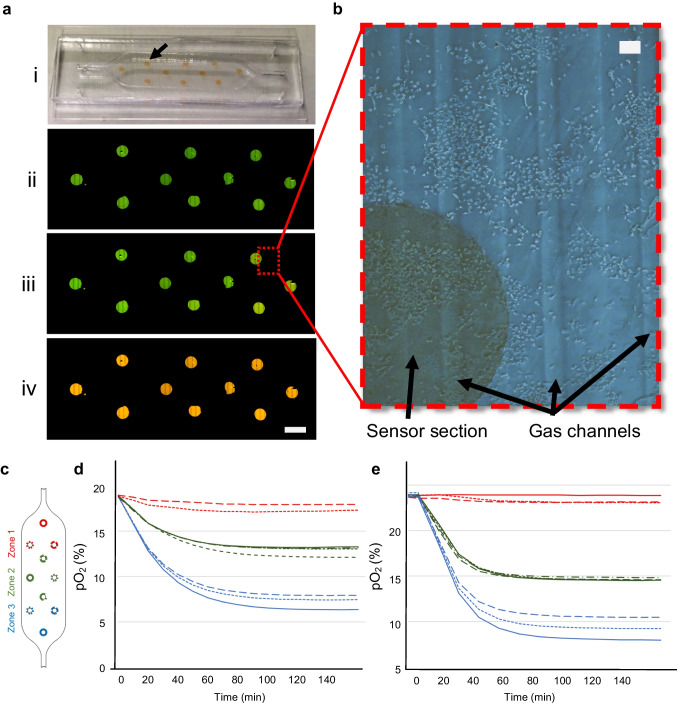
Oxygen measurement experiment conducted on the ZoC. **a-i** Displays the device equipped with ten distributed sensor sections across the culture area prior to measurements. An arrow indicates one of the sensors in the culture chamber. **a-ii** Shows fluorescence microscopy images of the sensors in the device at 19% percent oxygen, where the PtTFPP dye is strongly quenched, making the sensors appear green. The scale bar is 3 mm. **a-iii** displays the sensors of a cell-containing device during an oxygen gradient experiment, approximately at t = 120 min. In contrast, **a-iv** shows the sensors at 0% oxygen after the introduction of the sodium sulfate oxygen scavenger solution, allowing the PtTFPP dyes to emit light without quenching, resulting in the sensor sections appearing as orange, and providing a calibration point at the conclusion of the experiment. **b** Bright field image of the culture chamber with a sensor section during the experiment, showcasing cells scattered on the culture surface, with the parallel lines of gas channels visible in the background. The scale bar is 300 µm. The dynamics of oxygen concentration are shown through the readouts of each sensor sections in the device without cells **c** and with cells in the device **d**, respectively

To calibrate the time necessary for the gas channel network to equilibrate after the nitrogen gas is introduced, a sheet of sensor film was directly assembled to the gas layer, and the fluorescent timelapse signal was detected as readout, see Fig. 4c. Initially, the assembly is perfused with high oxygenated gas (19% oxygen). At t = 0, one of the gas inlet channels was switched to nitrogen gas (0% oxygen). Consequently, as time progresses the fluorescence of the sensor co-located with low oxygen side of the channel network is turning orange confirming the low oxygen concentrations in the region. The extracted fluorescence data from the indicated area in Fig. 4c (low oxygen area) can be converted to oxygen percentage level as a function of time (Fig. 4d). It is concluded that the sensor response time in this setting is 18 min. This temporal ratiometric shift is used in the numerical oxygen distribution simulations of the fully assembled device, where the cell chamber is also considered.

For the numerical dynamic oxygen distribution simulations, we introduced high (19%) and low (0%) oxygenated gases into the initially oxygen-saturated (19%) gas channel of the device (as in the calibration experiment mentioned above). The simulations were conducted using numerical models considering the location of the sensor sections used for ratiometric measurements (see Sect. 2.3 for details). Each of these locations represents a sensor section and serve as the regions of interest (ROI) (Fig. 4e). For each time step during the simulation, the average oxygen concentration in each ROI was plotted against time (Fig. 4f). A further refined scenario where the cells’ oxygen consumption was included is presented (Fig. 4g). Regardless of whether the cells’ oxygen consumption is considered in the simulation, oxygen concentrations diverged and reached distinctly separated zones already within a few minutes, stabilizing at around 30 min. Importantly, when including the cells’ oxygen consumption in the simulations, oxygen concentrations were notably lower, particularly in zone 3 where the concentrations were the lowest.

### Measurements of oxygen profiles in the ZoC device

Figure 4a-i depicts the ZoC device with integrated oxygen sensor sections, while Figs. 4a-ii and 4a-iii show the culture chamber with these sections visualized using fluorescence microscopy. Initially, the entire device was stabilized at 19% oxygen, indicated by the green fluorescence of the sensors across all regions as shown in the Fig. 4a-ii. During the experiment, as gases with 19% and 0% oxygen flow on either side of the device, a gradient forms, causing the sensor at the outlet (right side in Fig. 4a-iii) to shift in color towards orange. At the end of the experiment, the oxygen scavenger sodium sulfite was introduced into the culture chamber, causing a global decline in oxygen concentration to 0%. This change is observable through a color shift in the sensor sections, turning them orange (Fig. 4-iv). Figure 4b provides an additional bright field image of the culture chamber during the experiment, showcasing cells scattered on the culture surface alongside a portion of a sensor section beneath, with the further underlying parallel gas channels visible in the background.

To support the simulation results, ratiometric oxygen concentration measurements were conducted using sensor sections under two scenarios: with and without cells. The results of these measurement are presented in Figs. 4c and 4d, respectively. In both scenarios, all three zones reached distinct oxygen concentration equilibrium. Zone 1 maintained a normoxic condition at 19%, consistent with the concentration in the cell incubator, whereas zones 2 and 3 reached lower equilibrium levels. Notably, in zone 2, the oxygen concentration was approximately 12–14% in the absence of cells and 10–11% with cells present. In zone 3, there was a similar difference between the scenarios, with concentrations ranging from 6–8% in cell-free experiment and 3–5.5% with the cells in the device.

### Phenotype response to the ZoC oxygen gradient

**Fig. 6:**
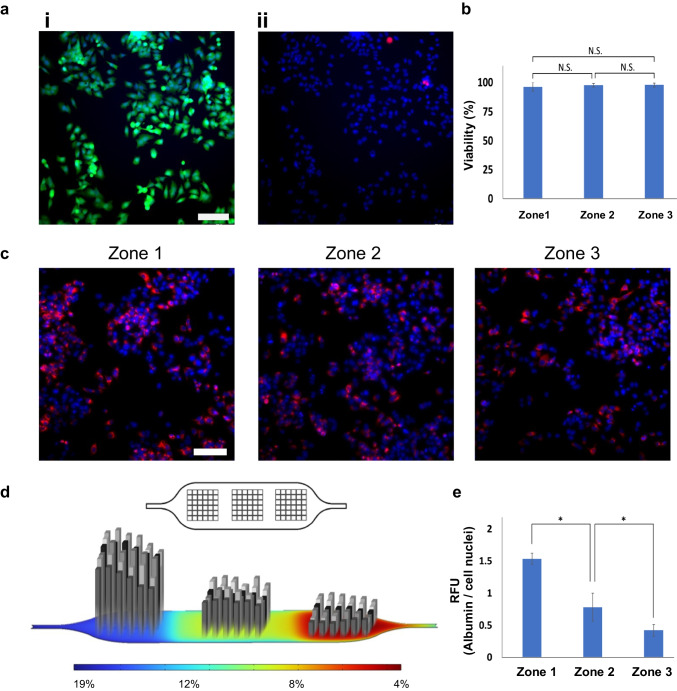
Quantitative assessment of HepG2 cell culture viability and functionality within the ZoC device 26 h post seeding. **a** Fluorescence microscopy images acquired from zone 3 show cells stained with the cell nuclei dye (Hoechst, blue) and the cytosolic live cell marker (calcein-AM, green) **i**, as well as the dead cell marker (ethidium homodimer-1, red) **ii**. **b** Presents the quantitative viability data for the three zones from three separate experiments. **c** Visualization of synthesized intracellular albumin (in red) and cell nuclei (in blue) using fluorescence microscopy, with representative images shown from each zone. Quantification of intracellular albumin levels involves measuring the red signal intensity normalized to the blue signal, presented as intensity per image within each zone **d** or as the average result per zone **e**. Data is acquired from three separate experiments. The scale bar in both the live-dead and the albumin immunostaining images is 100 µm

To evaluate the viability of on-chip cultured cells throughout the experiment, we conducted live/dead analysis. The analysis was performed 24 h after initiating the gas flow in the gas channels under a flow condition of 0.5 µL/min (26 h post cell seeding). Representative fluorescence microscopy images of zone 3 are shown, with panel (i) displaying live cells in green and panel (ii) showing all cell nuclei in blue, with dead cells highlighted in red, demonstrating high viability (Fig. 6a). Remarkably, over 95% of the cells maintained their viability across all three zones, indicating robust cell survival under these culture conditions (Fig. 6b). Cell viability is illustrated for each zone, with zone 1 exhibiting the highest oxygen levels and zone 3 the lowest.

Albumin expression was detected through on-chip immunofluorescent staining in the cells, followed by imaging and quantification. Representative fluorescence microscopy images from the three zones display albumin expression (red) and cell nuclei (blue) within the device after the 26-h experiment (Fig. 6c). Albumin production by the cells in all three zones was quantified based on the scanned imaging sites, as depicted in Fig. 6d. Each data point in Fig. 6d corresponds to an imaging position within the cell culture chamber, with 36 imaging positions allocated per zone. The albumin production is highest in the periportal region (zone 1) and lowest in the pericentral region (zone 3), corresponding to the highest and lowest oxygen tension levels, respectively. Albumin production in the intermediate region (zone 2) falls between these two. The cumulative data for each zone, demonstrate significantly different albumin expression levels that correlate with the oxygen concentrations (Fig. 6e).

## Discussion

We have engineered a microfluidic-based *in-vitro* cell culture device, termed the ZoC device, to replicate hepatic zonation. Initially, we designed, simulated, and fabricated a user-friendly device capable of generating and maintaining an oxygen gradient within a cell culture chamber. Next, we integrated oxygen sensors into the device to monitor the generated gradients and validate the simulated results. Finally, we verified the formation of zonation in cultured cells within the device by quantifying albumin production, which exhibited correlation with oxygen concentration, similar to the behavior observed in hepatocytes within sinusoids *in-vivo*.

Tissue oxygen concentration *in vivo* is influenced by various factors such as metabolism, local blood flow, and tissue thickness (Secomb 2017). For instance, in the liver acinus, oxygen levels vary significantly, ranging from 8.4% to 9.1% in the periportal area to as low as 4.2% to 5.0% in the pericentral zone (Kietzmann 2019). Traditional cell culturing approaches fail to replicate the structural complexities of the liver, resulting in uniform oxygen distribution, typically dictated by the incubator environment. Hence, the cultured cells might experience a too high oxygen concentration, not necessarily mirroring the oxygen concentration *in vivo*.

Furthermore, routine handling procedures of cell cultures such as medium exchange can disrupt oxygenation (Place et al. 2017), leading to limited control over oxygen levels during culture. Although oxygen is a key driver of hepatocyte zonation, nutrient gradients (including but not limited to glucose, hormones and cytokines) also contribute to this phenomenon (Kietzmann 2017). In conventional cultures without perfusion, nutrients are constantly depleted by the cells and are only replenished when the media is changed. This leads to fluctuations in nutrient and oxygen availability, which undermines their reliability and significance. Microfluidic technology offers a solution by providing precise control over the cellular microenvironment, allowing custom regulation of oxygen levels.

In our microfluidic-based ZoC device, the dual-layer design and the high gas permeability of PDMS facilitate oxygen diffusion from the gas channel through the overlaying PDMS layer into the cell chamber, creating three distinct oxygen concentration zones that mimic *in-vivo* zonation. This setup allows for experiments using the same cell culture batch within the same device, but exposing cells to different oxygen levels, thereby enhancing significance and reducing inter-experimental variations. In addition, conducting parallel culture experiments under varied oxygenation conditions would be inefficient in terms of time and resources. Furthermore, the possible paracrine effects of cells in different zones are completely overlooked when studies are conducted in separate culture flasks or dishes. Conversely, our ZoC device provides three distinct oxygen concentrations within a single device, along with controlled shear stress, utilizing nitrogen and ambient air to achieve desired oxygen levels. As cells are exposed to decreasing oxygen and nutrient levels sequentially along the culture chamber length, downstream cultured cells detect metabolites secreted by upstream cells. Therefore, the device is well-suited for studying cell communication between specialized cells.

In our ZOC device, we successfully created and optimized oxygen and nutrient gradients that align with each other, closely replicating the native state of the main driving forces of the zonation found in the liver acinus. In a similar work by Tonon et al. (Tonon et al. 2019), using one nitrogen gas inlet and the incubator air, both oxygen and nutrient gradients are generated to drive cell differentiation on-chip. Nevertheless, the oxygen gradient in their design is oriented perpendicular to the medium flow (and the nutrient gradient), differing from the *in vivo* liver acinus, where both gradients run parallel along the sinusoid. This deviation from physiological conditions could influence the interpretation of experimental outcomes.

Establishing an optimal balance between a physiologically relevant glucose distribution and minimal shear stress while effectively supplying oxygen across the three device zones is essential. Experimental parameters were optimized using CFD simulations prior to device fabrication. We selected a flow rate of 0.5 μL/min to maintain low shear stress (around 2.8 × 10^–6^ Pa), aligning with the suggested range for enhanced hepatic cell functionality (Rashidi et al. 2016). While higher flow rates also maintain suitable shear stress (Fig. 4b) (Tanaka et al. 2006; Tilles et al. 2001), our choice aimed to generate a glucose gradient reflecting fasting glucose concentration in liver sinusoids (4.4–6.7 mol/m^3^ (Edgerton et al. 2006)), evident in our ZoC. Our CFD results demonstrate a 24% decrease in glucose concentration along the ZoC flow direction, from 5.5 to 4.2 mM, emphasizing how altering flow rates can affect nutrient gradients (Fig. 4a). The nutrient gradient formation is influenced by cell type and metabolic rate, allowing our device to introduce media at various flow rates for cell type-specific glucose gradients. (For a detailed discussion on using the CFD analysis on organ-on-a-chip systems please refer to our previous study (Mahdavi et al. 2024)).

Our simulation model assumes PDMS saturation with oxygen at the high-oxygen gas channel due to the significantly higher gas flow rate exceeding saturation thresholds, an approach that conserve computational resources. We adopt a partition coefficient of 10 for oxygen transfer between culture medium and PDMS, as per previous work (Shiku et al. 2006). However, experimental observations indicate slower equilibration times up to two hours, possibly due to the interface effect or protein build-up on chamber walls, not included in our model assumptions. To address potential equilibration time in the gas tubing and gas channel network, we conducted dedicated experiments mirroring oxygen measurements, refining the simulation model (Fig. 4c-d). Furthermore, in our experimental oxygen measurements, the sensors are positioned beneath a thin, spin-coated PDMS layer of approximately 10 µm, which could potentially introduce additional latency. However, given the thinness of the PDMS layer, this effect is considered neglectable. Notably, we did not account for gradual protein sedimentation on the chamber walls in our model assumptions. Over time, protein accumulation on the PDMS surface may reduce its effectiveness in supplying oxygen to the cells, thereby decreasing oxygen transfer at the interface (Zanzotto et al. 2004). This protein build-up primarily impacts long-term PDMS-based microfluidic cell cultures and likely contributes to the slower equilibration observed in our experiments. However, for short-term experiments like ours, this effect is less concerning.

The equilibrium oxygen concentration in each zone is primarily determined by the gases supplied to the gas layer. However, because the device is not sealed against the ambient environment, some gas exchange also occurs between the environment and the cell chamber. This exchange is minor compared to the impact of cellular oxygen consumption. Both simulations and experiments demonstrated that a sufficiently large number of cells can significantly affect the equilibrium oxygen concentration.

On the fabrication side, maintaining a consistent thickness of both gas and cell layers is essential for achieving reproducible oxygen gradients as thicker devices create stronger diffusion barriers, potentially leading to inconsistencies in oxygen concentrations across different experiments. While creating gaseous insulation around the upper and lateral regions of the device could potentially create an even more regulated oxygen environment (Esch et al. 2014; Rafat et al. 2009; Wasay & Sameoto 2015), in this study, we intentionally relied on the ZoC device being exposed to ambient air thus prioritizing the user-friendliness of the setup. To attain the intended oxygen gas distribution used in this study, it was essential to maintain the total height of the gas channel network within 2 mm ± 0.3 mm. Thus, by simply adjusting the thickness of each PDMS layer, the oxygen concentration distribution within the cell chamber can be controlled and easily customized for each experiment, allowing for flexibility across a range of applications. Additionally, the chip allows for easy access to the cells, and the device fabrication requires one less assembly step compared to insulated counterparts. The separation of the gas channels from the culture chamber mitigates contamination risks, allowing the use of non-sterile gas sources without compromising experimental integrity.

For our experimental oxygen measurements, we utilized circular sensor sections scattered across the culture surface as probes. This approach was chosen to avoid significant alterations of oxygen levels within the chip, which would have occurred if we had used an entire sensor film due to the higher oxygen diffusion properties of PDMS compared to PS (sensor matrix). Additionally, using the entire sensor film would have compromised fluorescence cell imaging due to the presence of spectrally overlapping oxygen sensing dyes. The sensor sections were placed at zone boundaries where discrepancies were likely due to proximity to the uninsulated device border or zonal changes, and measurements confirmed a relatively uniform oxygen distribution within the zones with a concentration variation of only 2%−3% within the zones. The most significant concentration variation occurred in the low oxygen region (zone 3), probably because of the largest oxygen concentration difference between the zone 3 and the ambient atmosphere.

During a cell-free experiment, we discovered that prolonged illumination (455 nm) could cause bleaching of sensor sections, impairing their functionality. The issue was identified after one sensor section in a cell-free experiment was accidentally exposed illumination during the entire experiment for a few hours, causing an abnormal oxygen readout of well above 19%. The affected sensor flake from the experiment was hence omitted from the dataset (Fig. 4c).

Our study demonstrates a high cell viability across all three oxygen zones in the ZoC, validating the minimally invasive approach to oxygen tension modulation within our device, indicating its capability to support cell culture under varying oxygen conditions. The high viability is attributed to the passive diffusion of oxygen through the PDMS material, eliminating the need for neither oxygen suppliers nor scavengers. Remarkably, cells remained highly viable even in zone 3, which had the lowest oxygen level, suggesting the device capability to support cell culture under varying oxygen conditions.

On-chip immunostaining for quantifying albumin production provides distinct advantages over conventional ELISA-based measurements. While ELISA is the gold standard for measuring secreted albumin protein in liver chips (Chen, Miller, & Shuler, 2018; Du et al. 2021; Vernetti et al. 2016; Ya et al. 2021), in our ZoC, it would provide albumin measures from the outlet media, representing only an average of production from the three zones. In contrast, our approach visualizes the intracellular albumin production patterns locally within the three zones. By fixing the cells, staining them for albumin content, and acquiring an array of 36 fluorescence microscopy images for each zone, we achieved high-resolution quantification of local albumin production. This ensured obtaining data from within each distinct oxygen zone rather than from areas lying in the borders between two zones. Here, the positioning of the arrays was guided by the CFD analysis. Ultimately, the achieved three distinct oxygen zones demonstrate successful functionality, which was supported by the CFD analysis.

Even in standard incubators operating under normoxia in 19% oxygen, it has been shown that high-oxygen-consuming cells like hepatocytes can decrease the oxygen concentration to hypoxic levels (Al-Ani et al. 2018; Place et al. 2017). However, we have demonstrated that our chip can maintain normoxic oxygen concentrations in the periportal zone (zone 1) even in the presence of cell culture. This can be attributed to the oxygen supplement from both bottom and top of the culture chamber. Even with the distinct production levels of albumin, confirming the zonation, we observed slightly higher albumin production on one longitudinal side of the device, likely influenced by the gas channel’s configuration. This configuration slightly impacts oxygen availability to cells at the chamber’s border, thus affecting albumin synthesis (i.e., the increased fluorescent albumin intensities in the corresponding images within each zone).

Finally, we believe our ZoC device offers several advantages, though there are also areas for improvement that should be noted. One commonly mentioned drawback of PDMS is its material adsorption properties. Despite being easy to work with, biocompatible, gas permeable, and optically transparent, PDMS can adsorb and even absorb small hydrophobic molecules (Toepke & Beebe 2006) and (Butler et al. 1997). This limitation not only affect the infusion of various on-chip staining dyes but may also hinder the treatment of cell cultures cultured in the ZoC device with known or candidate drug substances. If these substances adhere to the material rather than reaching the cells, consequently, the concentrations needed to elicit a significant cellular response may appear much higher than would be the case in another *in vitro* cell culturing setting. Therefore, identifying a material that does not exhibit such adsorption while maintaining the other advantageous properties of PDMS would enhance our device.

One notable advantage of PDMS is the ease of replication, provided a mold is available. In our case, creating the cell chamber mold is relatively straightforward, as 3D printers are widely accessible today. However, the gas channel network, which has much finer dimensions, is more challenging to fabricate without specialized soft lithography equipment. Although we have implemented measures to reduce complexities, such as oxygen insulation, the two-layer design still requires a multistep fabrication process, which adds to its complexity. Nonetheless, this ZoC design could potentially be adopted by larger, off-the-shelf microfluidic manufacturers to make the concept more accessible to the research community.

A final limitation is that this study using our ZoC device focuses solely on 2D culture, which is optimal for certain applications, such as endothelial cells. However, the ideal scenario would involve expanding this concept into a 3D setting. This transition, though, would require further evaluation of how 3D tissue affects gas distribution and may necessitate fine-tuning of the gas channel network and layer thicknesses.

## Conclusion

In conclusion, this study presents a novel microfluidic device designed to induce zonation in liver hepatocytes by establishing three distinct oxygen tension levels within the cell culture area. By incorporating a gradient of nutrients and oxygen, this device effectively mimics the physiological conditions of the liver microenvironment. Through simulations and ratiometric oxygen sensing, we validated the device's capability to establish an oxygen gradient along its length.

Quantification of albumin production by HepG2 cells using immunostaining and fluorescence microscopy revealed significant differences across the three oxygen zones, confirming the device's ability to elicit metabolic responses corresponding to varying oxygen concentrations. Despite minor discrepancies in oxygen readings due to sensor placement and border effects, the overall trend demonstrates the device effectiveness in modulating oxygen levels and studying cellular responses.

The simplicity and accessibility of the proposed device, which utilizes readily available laboratory equipment such as nitrogen line and miniature vacuum pump, contribute to its user-friendliness. This accessibility not only facilitates zonation research but also extends its utility to studies requiring oxygen gradients or hypoxic environments, eliminating the need for costly oxygen-modulating infrastructure. The relatively rapid attainment of equilibrium oxygen levels within approximately two hours, and the sustained high cell viability throughout the experiment further support the ZoC’s suitability for cell culture applications.

Finally, while initially tailored for liver zonation studies, the adaptability of this device suggests potential applications in diverse research areas where controlled oxygen tension or gradient environments are required. Our hope is that the ZoC device will assist researchers in identifying optimal oxygen levels that mimic *in vivo* conditions, ensuring the health and functionality of sensitive cells such as primary cells and stem cells. The flexibility of this technology opens new avenues for exploration in fields ranging from tissue engineering to cancer biology, where precise oxygen modulation is critical for understanding cellular behavior and disease progression. This work lays the groundwork for future investigations utilizing the accessibility of oxygen-modulating microfluidic devices for enhanced physiological relevance in cell biology research, and potentially challenging the current cell culturing paradigm.

## Data Availability

Data is provided within the manuscript.
